# Music Performance As an Experimental Approach to Hyperscanning Studies

**DOI:** 10.3389/fnhum.2016.00242

**Published:** 2016-05-25

**Authors:** Michaël A. S. Acquadro, Marco Congedo, Dirk De Riddeer

**Affiliations:** ^1^Centre National de la Recherche Scientifique, Gipsa-Lab-DIS, University of Grenoble-AlpesGrenoble, France; ^2^Dunedin School of Medicine, Otago UniversityDunedin, New Zealand

**Keywords:** hyperscanning, music, brain and body coupling, EEG, social interaction, ecological paradigm

## Abstract

Humans are fundamentally social and tend to create emergent organizations when interacting with each other; from dyads to families, small groups, large groups, societies, and civilizations. The study of the neuronal substrate of human social behavior is currently gaining momentum in the young field of social neuroscience. Hyperscanning is a neuroimaging technique by which we can study two or more brains simultaneously while participants interact with each other. The aim of this article is to discuss several factors that we deem important in designing hyperscanning experiments. We first review hyperscanning studies performed by means of electroencephalography (EEG) that have been relying on a continuous interaction paradigm. Then, we provide arguments for favoring *ecological paradigms*, for studying the *emotional component of social interactions* and for performing *longitudinal studies*, the last two aspects being largely neglected so far in the hyperscanning literature despite their paramount importance in social sciences. Based on these premises, we argue that *music performance* is a suitable experimental setting for hyperscanning and that for such studies EEG is an appropriate choice as neuroimaging modality.

## Introduction

The behavior of human in groups is extremely complex, depending on both the single entities forming them, as taken individually, and on their dynamical interactions, which is the product of the context and of specific combinations of individuals. The aim of social neuroscience is to uncover the neural underpinning of the human social behavior from both an individual and a dynamical perspective. The question is how the brain *per se* is prepared to such behavior, thanks to specific *a priori* characteristics of the brain and how it may change its functioning in a social interaction, that is, whether there exist *emerging brain coupling characteristics* (Ochsner and Lieberman, 2001; Stephens et al., 2010; Frith and Frith, 2012; Hasson et al., 2012).

Prior to the onset of language, the primary means by which infants can communicate with others in their environment, including caregivers, is by ‘reading’ faces. It is important for an infant not only to discriminate familiar from unfamiliar individuals, but also to derive information about the individual’s feelings and intentions. The ontogenetical tuning of the developing brain to social signals of emotions therefore starts at birth with the anatomical emergence of a network (amygdala, orbito-frontal cortex, superior temporal sulcus, fusiform gyrus) that permits the recognition of different emotional facial expressions as from the age of 5–7 months (Leppänen and Nelson, 2009).

In adults, two distinct networks, the mirror neuron system (MNS) and the mentalizing network (MENT) may contribute to social interaction. The MNS is supposed to involve preconscious mechanisms which underlie/facilitate sharing (and mimicry) of others’ behaviors and internal states, whereas the mentalizing system is involved in conscious, deliberative process through which inferences can be made about others’ bodily and affective states, beliefs, and intentions (Christov-Moore et al., 2014). The MNS is hypothesized to permits prediction of other’s intentions (Aglioti et al., 2008; Southgate et al., 2009; Kilner and Friston, 2014; Maranesi et al., 2014). Its anatomical substrate in humans, based on a meta-analysis of 125 functional magnetic resonance imaging (fMRI) studies appears to consist in the inferior parietal lobe, ventral premotor cortex, inferior frontal gyrus, dorsal premotor cortex and superior parietal lobe (Molenberghs et al., 2012). The mentalizing network consists of the superior temporal sulcus (STS), as well as the anterior cingulate cortex, temporo-occipital junction (BA37), temporal pole and the amygdala (Molenberghs et al., 2012). Mentalizing may have arisen in part as a form of contextual control for mirroring (Christov-Moore et al., 2014). Accurately discerning the internal states of others, as well as inferring intentions from observed behavior, rely on the interaction between mirroring and mentalizing processes (Zaki and Ochsner, 2012). The reason is that interactions between mirroring and mentalizing may allow individuals to revisit past experience and behavior, predict the consequences of their own behaviors, both for themselves as well as for others, and to selectively share in the behavior and affective states of others in response to context (Christov-Moore et al., 2014). We note that while the literature on these two networks is very large, recent research challenges the existence of the MNS in humans (Lingnau et al., 2009).

The study of *emerging brain characteristics* has appeared recently thanks to the advent of the simultaneous brain scanning of two or more individuals while they are interacting, a technique known as *hyperscanning* (Montague, 2002; Dumas et al., 2010; Astolfi et al., 2011a; Babiloni et al., 2012; Cui et al., 2012; Sänger et al., 2012). So far, hyperscanning studies have employed, as neuroimaging modalities, fMRI (Montague, 2002; King-Casas et al., 2005; Tomlin et al., 2006; Redcay et al., 2010; Krill and Platek, 2012; Lee, 2015), electroencephalography (EEG: Babiloni et al., 2006; Yun et al., 2008; Lindenberger et al., 2009; Dumas et al., 2010; Fallani et al., 2010; Astolfi et al., 2011b; Kawasaki et al., 2013; Müller and Lindenberger, 2014) and near-infrared spectroscopy (nIRS: Funane et al., 2011; Cui et al., 2012; Dommer et al., 2012; Holper et al., 2012; Jiang et al., 2012; Cheng et al., 2015). For these studies, several authors have recommended the use of socially and phylogenetically relevant stimuli such as human faces (Haxby et al., 2000; Gamer and Büchel, 2009) and gaze perception (Pelphrey et al., 2004). These kinds of social cues can change very rapidly (Littlewort et al., 2006). The same is true for emergent behavioral patterns in role taking (Reed et al., 2006) or leading (Van Knippenberg, 2011). While different neuroimaging modalities provide complementary information about brain functioning due to their different spatial and temporal resolution (Hari and Parkkonen, 2015), only EEG can capture dynamical changes at the 10s of millisecond scale, allowing the study of social dynamics at the temporal scale they occur. Furthermore, EEG recording can be carried out in ecological situations. Indeed, a fundamental factor in social behavior is the context. The use of experimental designs allowing an ecological interaction between participants has been warmly recommended by many authors (Hari and Kujala, 2009; Di Paolo and De Jaegher, 2012; Schilbach et al., 2012).

**FIGURE 1:**
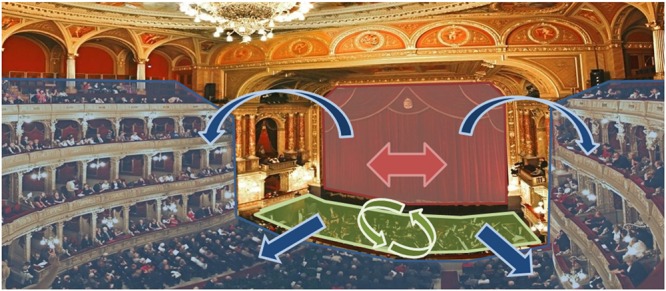
**Example of ecological activity presenting the three levels of interaction.** The three levels of interaction are present during an opera performance. Actors (red zone) interact with each other in a *turn-based* fashion, taking turns in signing and acting (red arrows). The orchestra (green zone) accompanies the play, each musicians *continuously interacting* with one another (green arrows). The public (blue zone) watches the play, sitting in an *observation condition*, actors and musicians being two audio and visual stimuli (blue arrows).

**FIGURE 2:**
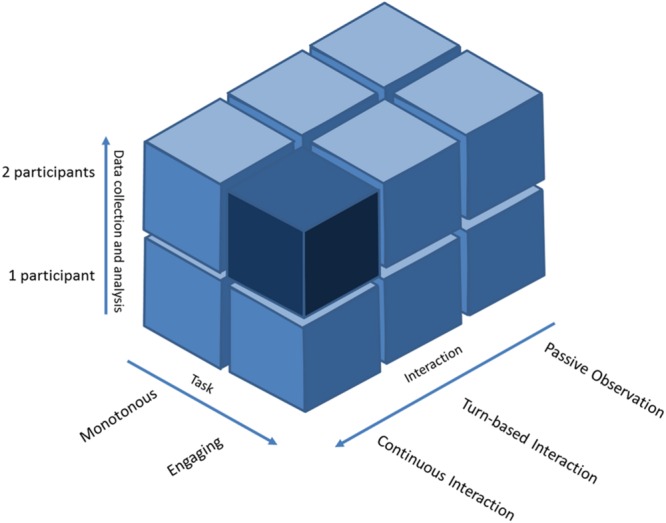
**Representation of the experimental ‘landscape’ of the two-person neuroscience.** Rearranged from Schilbach et al. (2012). The cubes represent different categories of experiment in the two-person neuroscience according to three axes: (1) data collection and analysis from 1 to 2 persons (hyperscanning); (2) participant’s engagement during the experimental task from detached to fully engaged (emotions, ecological significance, longitudinal experiment); (3) degree of interaction from passive observation, through turn-based interaction, to continuous interaction. The darkest area represents the “dark matter” of social neuroscience that has been the least explored. Music performance as a hyperscanning paradigm could help unravel this area.

Social interaction may be categorized according to three different levels: *observation condition, turn-based interaction* and *continuous interaction* (**Figure 1**). Each category has its own role in deciphering the physiological markers of social interaction, but the continuous interaction has a privileged role. In the *observation condition*, an isolated individual is confronted to social cues. It has been extensively investigated since the birth of social neuroscience and has been instrumental for MNS and MENT studies (Adolphs, 2002a; Decety et al., 2002; Wicker et al., 2003; Pelphrey et al., 2004). In the *turn-based interaction*, two or more individuals socially interact in specific tasks requiring a turn-based interaction mode. It has been investigated more recently thanks to the hyperscanning technique for social activities such as card playing (Babiloni et al., 2006), trust games (Montague, 2002; King-Casas et al., 2005; Fallani et al., 2010) and speech (Kawasaki et al., 2013). In the *continuous interaction*, two individuals dynamically interact with very low constraints, either by acting at the same time or by arbitrarily switching roles at fast pace. The advantage of this social interaction is that it mirrors daily human behavior, such as conversation (Jiang et al., 2012). Thus it enables the study of natural behavior. This paradigm has been employed only in a few studies so far (Babiloni et al., 2006, 2012; Astolfi et al., 2011a; Müller et al., 2013; Müller and Lindenberger, 2014), partly because of the difficulty to design a socially ecological experiment with strong experimental control, the restricted use of neuroimaging modalities capable of recording events on the millisecond timescale, as well as the unavailability of appropriate methods of hyperscanning data analysis. **Figure 2** shows the experimental landscape of the two-person neuroscience according to the types of interaction we have just described.

The aim of this article is to discuss several factors that we deem important in designing hyperscanning experiments. We first review EEG-hyperscanning studies that have been relying on a continuous interaction. Then, we provide arguments for favoring *ecological paradigms*, for studying the *emotional component of social interactions* and for performing *longitudinal studies*, the last two aspects being largely neglected so far in the hyperscanning literature despite their paramount importance in social sciences. Based on these premises we argue that *music performance* is a suitable experimental setting for hyperscanning and that for such studies EEG is an appropriate choice as neuroimaging modality.

## Eeg-Hyperscanning Studies of Continuous Interactions

In the study of Tognoli et al. (2007), participants performed an in phase finger-tapping task, with or without visual feedback. Their high-resolution spectral analysis of EEG activity revealed an oscillatory component (phi1, phi2: the phi complex) located on the right centro-parietal cortex in the frequency band 8–12 Hz. This component was found to be sensitive to both independent and synchronized movements with phi1 promoting independent behavior and phi2 favoring coordinated behavior (Tognoli et al., 2007).

Naeem et al. (2012) replicated the previous experiment with finger movement adding an anti-phase condition. They found similar results as Tognoli et al. (2007), plus a modulation of the mu frequency band (10–12 Hz) differentiating intent to move the finger in phase or anti-phase (Naeem et al., 2012). These studies provided preliminary results on *a priori* brain mechanisms allowing motor coordination in a continuous interaction, but did not investigate the inter-brain dynamics and the brain networks at play during the synchronous tapping task.

The study of Yun et al. (2012) employed the idea of implicit synchronization when physically interacting with another human. The experiment consisted of three phases. First, participants sitting face to face had to stretch one arm toward each other (right arm vs. left arm). They had to look at each other’s index finger while trying to stay as still as possible. Then, one leader was randomly chosen. His task was to make arbitrary motions with his hand. The partner had to reproduce the motions as precisely as possible. Finally, participants were asked to come back to the first phase and remain as still as possible. The behavioral analysis showed that participants implicitly synchronized their hands with each other, even more so during the last phase of “stillness” as compared to the first phase. The authors used the Phase Locking Value (PLV), a measure that can provide an indication of short-range neuronal synchronies that can be interpreted as subserving ‘perceptual binding’ between adjacent brain regions. The PLV may also describe long-range synchronization patterns among widely separated areas that could contribute to cognitive mechanisms in the same brain or even in different brains (i.e., social bonding; Lachaux et al., 1999). They found increased phase synchronization in theta (4–7 Hz) and beta (12–30 Hz) frequency bands between participants in the inferior frontal gyrus, anterior cingulate, parahippocampal gyrus, and post-central gyrus following the second phase. The authors interpreted these results as an increased coupling between one’s own introspective thinking and the other’s representations, in addition to the detection of visual cues dependent on social context. This study demonstrates that a simple social interaction activity such as tracking the movement of another person changes the functioning of the brain in a way that cannot be ascribed to each individual taken separately.

Along the same lines, Dumas et al. (2010) set up a dual video feedback system so that participants could see the hands of their partner. The participants were to either imitate the other’s hands movement when they felt the urge to, or a designated leader had to move and the other had to try to follow. Using a PLV analysis, they demonstrated increased between-brain phase synchronization in the frequency bands mu (9–11 Hz), beta (13–30 Hz), and gamma (31–48 Hz) in the right centro-parietal areas of the two participants during period of behavioral synchronies as compared to unsynchronized periods. More importantly, no difference was found between imitation and non-imitation conditions, discarding the possibility that the inter-brain synchronization was due exclusively to a similarity in the performance and perception of gestures. Finally, the synchronization patterns in the higher frequency bands (beta and gamma) were asymmetrical between model and imitator’s regions. The authors argued that this asymmetry could be seen as a brain to brain top–down modulation reflecting the differential roles attributed (spontaneous or imposed) in leading or following.

In the study by Astolfi et al. (2011a), an airline pilot and co-pilot where scanned simultaneously during the takeoff, flight and landing of an airplane. The entire experiment was conducted inside a simulator for safety reasons. They employed partial directed coherence (PDC; Baccalá and Sameshima, 2001) as a directed measure of phase synchronization between individuals. The aim of this measure is to simultaneously quantify the degree of linear interdependency between multiple signals in the frequency domain, and because it is directed, it may help to describe asymmetrical relationships between participants (i.e., leader-follower). The experiment showed significant increase in the power spectral density (PSD) in the theta frequency band (3–7 Hz) in frontal electrodes (F3, Fz, and F4), as well as a suppression of the PSD in the alpha band (8–12 Hz) in parietal electrodes (P3, Pz, and P4) when the task required a strong cooperation between the captain and his first officer (i.e., takeoff and landing). The strongest connections revealed by the PDC measure involved frontal electrodes, and were directed from the first officer toward the captain, in accordance with the task of the first officer to read and control instrumentation while the captain controls the plane. During phases not requiring extensive interactions between pilots (i.e., flight phase), little to no inter-brain phase synchronization was found. This result suggests that in a realistic situation a series of activities with a cooperation goal elicits functional connectivity between the brains of effectors, here pilots. This study is very interesting because it employs an ecological experimental setup, although without a strong emotional component.

Dodel et al. (2011) proposed a novel approach to study the dynamics of interpersonal coordination based on singular value decomposition (SVD). During a task involving a simulated team fighting game, it was demonstrated that participants of the same team had their EEG activity evolving along a common sub space with a dimensionality and signature specific of their coordination. In addition, the expertise of a team had an impact on the intrinsic dimensionality of their sub space. An expert team had less dimensions than a novice; which is well in line with recent research on interpersonal synergies (Riley et al., 2011).

Lindenberger et al. (2009) carried out an experiment on guitar duos playing a melody. Authors used two measures of phase synchronization that they named Phase Locking Index (PLI) and inter-brain phase coherence (IPC); the former measured the consistency of phase at one electrode across trials, while the latter measured the consistency of the relative phase at two electrodes from two different brains across trials. They found significant within and between brains increase in phase synchronization during (i) periods of preparatory metronome tempo setting between 2 and 10 Hz, with the maximum in the theta frequency range (4–7 Hz), and (ii) after play onset between 0.5 and 7.5 Hz, with the maximum at 3.3 Hz. In this experiment, the two guitarists are playing the same melody at the same tempo, and are immersed in a very similar and highly coordinated sensorimotor flow, which by itself may explain the fact that the sensorimotor areas are synchronized (Chatel-Goldman et al., 2014). In addition, they were behaviorally synchronized on the same tempo with the use of an external metronome.

**FIGURE 3:**
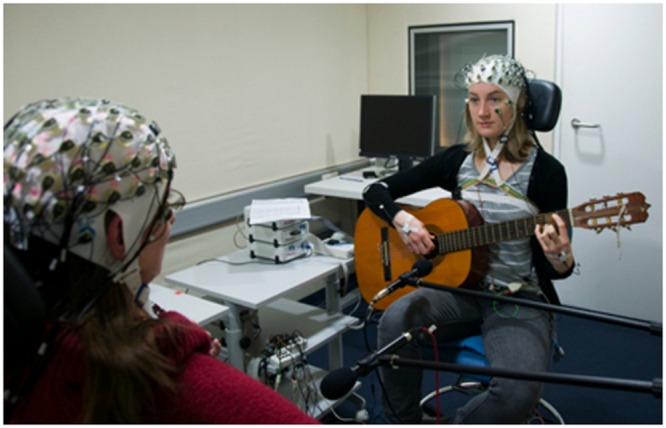
**Advantages of EEG as a neuroimaging modality.** From Sänger et al. (2012). The experiment from Sänger et al. (2012) is an appropriate example when it comes to studying social interaction in an ecological way with a socially engaging task. In this experiment, guitar players were able to see each other’s face, enabling them to perceive each other’s gaze and facial expressions, crucial social cues in human interactions (empathy, theory of mind). Additionally, EEG as a neuroimaging modality is moderately hindering musicians’ movements compared to other modalities such as fMRI and MEG, allowing them to interact in a more ecological manner. Finally, the task choice (music performance) is pertinent for many reasons that we will discuss, but mainly because it is socially relevant: practicing music with someone is a common activity.

In order to verify that the brain synchronizations were not only due to similar sensory input and/or motor output, Sänger et al. (2012) carried out a similar experiment by changing the guitarists music sheets so they did not exactly play the same partition, while keeping the need for musical coordination. Examining the previously found frequency bands with the same PLI and IPC measures as well as graph theory to investigate intra- and inter-brain networks (Bullmore and Sporns, 2009), they found increased phase synchronization in networks within and between brains in the frontal and central electrodes during periods requiring high musical coordination (**Figure 3**).

A more recent study by the same authors (Sänger et al., 2013) explored the effect of role in a guitarist duet on brain coupling using a measure of time-lagged phase synchronization, the Integrative Coupling Index (ICI), as well as graph theory. One musician was designated leader and was requested to manage the playing tempo as well as bringing the follower in at play onsets. After determining the graph’s architecture including both participants’ brains, researchers showed that synchronization from the leader’s frontal nodes to the follower’s brain was stronger in the high alpha (12 Hz) frequency band during time segments of 500 ms around coordinated play. Authors argued that this demonstrated the importance of the leadership on the time parameter of their joint action. This result directly echoed the work of Dumas et al. (2010) on the synchronization of hand movements.

A recent research carried out using a synchronized finger-tapping task confirmed this asymmetrical trend in leader-follower interaction (Konvalinka et al., 2014). They found that frontal alpha-suppression could differentiate roles, being more enhanced in leaders than followers. According to the authors the differentiation may be due to the fact that leaders must allocate more resources to self-processing to attend their own beat, rather than monitoring the output of their partner (Konvalinka et al., 2014).

Another paper carried out by Müller et al. (2013) involved musical improvisation; one of the concerns about previous studies was that a multiple trial design constrained the range of observable behaviors, thus preventing a reliable investigation of real life events. The authors tried to overcome this limitation by designing a task where guitarists had to perform jazz improvisation for a few minutes. Additionally, this time they did not provide a metronome so that musicians were free to conjointly choose their preferred tempo. The data analysis was performed using ICI and graph theory. The results showed intra- and inter-brain connections within a hyper-brain network in all duets: the first, intra-brain, distributed across the entire cortex relying on high frequency band (e.g., beta: 14–28 Hz), and the second, inter-brain, involving lower frequencies (delta: 23 Hz; and theta: 5–7 Hz) and varying across time and musicians. Moreover, they found that some network proprieties were related to the musical role, melody and chords accompaniment during improvisation. The authors argued that the increase of connector hubs at low frequencies might point out to mechanisms enabling individuals to interact in coordination during temporally joint actions (i.e., jazz improvisation). This study is unique in the field of EEG hyperscanning in that the experimental setup grantees freedom of movement and decision (improvisation) while employing an engaging emotional task.

Babiloni et al. (2012) studied a saxophone quartet when playing “en ensemble.” Musicians had to go through four conditions: playing the musical piece, resting, watching video of their own collective performance’s video, and watching a control video. In addition to the four simultaneous EEG recordings, they administered a questionnaire measuring empathy. A source analysis was performed using the standardized low-resolution brain electromagnetic tomography (sLORETA; Pascual-Marqui, 2002; Congedo, 2006) and a cortical activation/deactivation index was computed as a task-related power decrease/increase (TRPD/TRPI) of EEG alpha rhythms (8–14 Hz). The results showed that the higher the empathy quotient in a musician was, the more one could observe a desynchronization of the alpha frequency band in the right 44/45 Brodmann area. Authors suggested that during the musician’s observation of his own performance, left ventral-frontal alpha desynchronization underlined global attention and emotional empathic processes. This study has the merit of introducing the idea of emotion and empathy interplay during activities requiring cooperation and behavioral coordination.

In a recent study led by Chatel-Goldman et al. (2014), researchers focused on the emotional aspect of social interaction by asking romantic partners to convey emotions through an uncanny channel: affective touch. In this study skin conductance, heart rate variability, respiration, as well as EEG, were simultaneously recorded. The romantic partner had to take each other’s hand and, with no eye-contact, try to transmit a positive or negative emotion. Researchers found that interpersonal romantic touch increased coupling of electrodermal activity between couples, regardless of the nature of emotion, and that physical touch induced reliable changes in the physiological states of romantic partners. Although, this study did not reveal significant results with EEG data, it has found interesting physiological results suggesting that touch alone can allow the emergence of a somatovisceral resonance between couples.

A second study on touch was carried out by Müller and Lindenberger (2014). They investigated romantic kissing in humans. Using graph theory and cross-frequency coupling (CFC; Canolty et al., 2006), the researchers examined whether the brains of romantic partners are more synchronized when kissing each other as compared to kissing their own hands or while performing an arithmetic task. The results showed some similarities with their previous study on guitar duets; they found a theta-alpha hyper-brain subnetwork between subjects indicated by all intra- and inter-brain connections including CFC from 5 to 10 Hz, but also 10 to 5 Hz. They also found a reliable correlation between kissing satisfaction and the inter-brain strength for 5 Hz oscillation nodes. This correlation was greater for the romantic kissing condition as compared to the control condition (own hand kissing). The authors suggested that theta phase synchronization could describe interpersonally coordinated voluntary actions, and bonding behavior. A summary of this review is provided in **Table 1**.

**Table 1:** Summary of devices, types of stimuli, types of interaction, and methods of analysis used in hyperscanning studies.

	Device	Experimental paradigm	Interaction	Method of analysis	Reference
Less ecological	fMRI x2	Trust game	Turn-based	Coherence	Montague, 2002
	NIRS x2	Button pressing, cooperation/competition	Turn-based: trials	Wavelet transform coherence	Cui et al., 2012
	EEG x2	Prisoner’s dilemma	Turn-based	Granger’s causality	Babiloni et al., 2007
	EEG x2	Chicken’s game	Turn-based	Directed transfer function, partial directed coherence	Astolfi et al., 2010
	EEG x2	Speech rhythm	Turn-based	Wavelet transform, cross-correlation analysis	Kawasaki et al., 2013
	EEG x2	Finger tapping	Continuous	Power spectra analysis (no inter-brain analysis)	Tognoli et al., 2007
	EEG x2	Finger movement	Continuous	Phase synchrony and behavioral analysis	Yun et al., 2012
	EEG x2	Hands movement	Continuous	Phase locking value	Dumas et al., 2010
More ecological	fMRI (no hyperscanning)	Natural movie viewing	Observation	Correlation of hemodynamic waveform	Hasson et al., 2004
	EEG x4	Card Game	Turn-based	Partial directed coherence	Babiloni et al., 2006
	EEG x2	Plane operating	Continuous	Partial directed coherence	Astolfi et al., 2011a
	EEG x4	Saxophone playing	Continuous	Frequency analysis (TRPD/TRPI) and correlation with empathy	Babiloni et al., 2012
	EEG x2	Guitar playing	Continuous	Phase Locking Index and inter-brain phase coherence	Lindenberger et al., 2009
	EEG x2	Romantic kissing	Continuous	Graph theory analysis, Adaptive Integrative Coupling Index	Müller and Lindenberger, 2014

*This table shows a representative sample of studies in hyperscanning according to the neuroimaging modality, the type of activity, and the type of interaction used. We indicate the method of analysis used to identify cerebral synchronization.*

## Ecological Validity

In contrast to the traditional method of neuroscience studying isolated individuals in an over-controlled environment (Hari and Kujala, 2009), the goal of social neuroscience is to use a paradigm revealing physiological markers of social interaction by studying humans in a more ecological environment (Schilbach et al., 2012). Ecological validity has a long history in psychology, with its meaning continuously debated by the community. Multiple definitions have been given (Scheidt, 1981). Their common denominator is the relationship between real-world phenomena and the investigation of these phenomena in experimental contexts. Three dimensions can be extracted from this notion: the nature of the setting, the stimuli and the response of participants (Schmuckler, 2001).

The first dimension, the *nature of the setting*, emerged from a discussion between Brunswik (1943) and Lewin (1943) on the environmental context of the research and its impact on the study. Brunswik was concerned that psychologists were more and more studying “narrow-spanning problems of artificially isolated proximal or peripheral technicalities of mediation which are not representative of larger patterns of life.” In order to avoid this reductionist approach, he advised to widen the scope of analysis from people to the situations of investigation (Brunswik, 1943). His concern and advice are increasingly relevant in studying human social interactions. Therefore, the *nature of the setting* refers to the extent to which the environment experienced by the subjects in an experimental investigation has the properties it is supposed or assumed to have by the experimenter (Bronfenbrenner, 1977). Although, the relevance of the environment is crucial when studying a particular behavior, it is often sacrificed for technical reasons. Recent studies show that hormonal influences may play an important role in modulating social behavior (Volman et al., 2011). Ecological situations, such as face-to-face interactions, could promote this channel of influence (Henckens et al., 2009). The second dimension of ecological validity, probably the best known, is the *stimuli employed*. According to the Gibsonian ecological approach (Gibson, 1966), the stimulus is not just an occurrence at the sensory receptor with no context or information about its source in the world; it is rather a rich nested event involving objects, surfaces, and their relations. Neisser (1976) stressed that ecologically valid stimuli involve a multimodal information pickup and integration within a continuous spatiotemporal flow, that is, an information requiring multiple senses to perceive and understand, while being presented as a whole in its natural context. Using ecological stimuli has been proposed often in recent years by the social neuroscience community (Schilbach et al., 2012). It has been argued that adding a second human being into the research box can be a viable solution to attain ecological validity (Hari and Kujala, 2009; Di Paolo and De Jaegher, 2012). The third and last dimension of ecological validity is the *response required from the participant*. The question is whether the subject’s response is natural and representative of the most appropriate behavior for the situation under investigation or if it is biased. This problem has been raised by Bronfenbrenner (1977) in the context of developmental psychology. The author complained that developmental psychology was becoming the “science of the strange behavior of children in strange situations with strange adults for the briefest possible periods of time.” This dimension is difficult to assess also because it interacts with the experimenter effect. One solution could be to act on the two previous dimensions by providing a socially meaningful situation with another human being as stimulus. The perception of each other’s gaze modulates socio-cognitive performance (Senju and Johnson, 2009). Di Paolo and De Jaegher (2012) call the disposition to engage with another human being in an interaction “*readiness to interact.”* They argue that a favorable environment and context would help recreate expectancies of social contingencies and anticipatory dispositions during communication (Jordan, 2009).

Maintaining ecological validity through the three dimensions described earlier usually trades off with experimental control. Attempts to reinforce one weaken the other and vice versa. Often the tradeoff can be optimized sacrificing ecological validity in some dimensions while maintaining it in the others (Schmuckler, 2001). A fine balance between experimental control and ecological validity is therefore a major challenge in designing hyperscanning studies.

## Emotional Component

Most of the contacts that we have with other people during a day or across our whole life are tinged with emotions (Dolan, 2002). The brain is constantly bombarded by simultaneous stimuli (Seeley et al., 2007), and behaviorally relevant or distinctive environmental events should be processed with priority (Dolan, 2002; Fecteau and Munoz, 2006; Seeley et al., 2007). From an evolutionary point of view, emotions evolved to create a priority mode for attentional perceptual processing (Dolan, 2002). Emotions are the constant “backdrop” accompanying us in our everyday life, greatly affecting our behavior and attitudes (Forgas, 2003). Our ability to manage emotions is related to the quality of social interactions (Lopes et al., 2004). The lack of emotional responsiveness may lead to irrational and even pathological behavior (Damasio et al., 1994). Recognizing emotions in another human being involves numerous psychological and neurological mechanisms (Adolphs, 2002b); emotions are an important component in acknowledging the others’ intentions and state of mind, even in young children (Harris et al., 1989; Leppänen and Nelson, 2009). The recognition of emotions in others likely depends on neural mechanisms that generate similar affect in the perceiver, allowing the sharing of emotional states among individuals (Preston and De Waal, 2002). A popular theory of empathy is known as the *shared network hypothesis*, according to which observing or imagining another in a particular affective state activates a representation of the same state in the observer with its autonomic and somatic responses (Lamm et al., 2011). Indeed, in a recent paper, Anders et al. (2011) demonstrated that during ongoing facial communication of affect by romantic partner scanned alternatively in an fMRI, emotion-specific information was encoded in similar brain networks in the two participants. Furthermore, they found that there was specificity in the temporal flow of the affect information from the sender to the perceiver. Indeed, when the information was delayed in the brain of the sender, the response was as much delayed in the brain of the perceiver (Anders et al., 2011). This phenomenon shows that emotional information was dynamically adjusted between the brains of each partner, and did not just carry “prototypical” information. This *emotional sharing* would then provide relevant information on the mental states of others, complementing non-emotional attributions. As such it is considered a precursor of empathy and can affect the motivation to communicate and cooperate with someone else (Preston and De Waal, 2002; De Vignemont and Singer, 2006). Emotional responses can be viewed as a catalyst for social behavior influencing our willingness to engage in relationships with others. In this direction, it has been recently found that emotions may enhance brain coupling between participants, such as increased theta-alpha (6–12 Hz) cross-correlation power between participants when listening to a familiar voice, or increased inter-subject correlation when experiencing negative valence emotions while watching a movie (Nummenmaa et al., 2012; Kawasaki et al., 2013). Experimental situations leading to emotional engagement could contribute to the understanding of social interactions, and improve the ecological validity of participants cooperating on a same task. As such, eliciting emotion experimentally is a relevant option for hyperscanning studies and allowing freedom to read emotional cues carried by the eyes or the face by using an appropriate imaging modality is important (i.e., EEG, fNIRS). However, most hyperscanning studies have not explicitly taken into consideration the emotional component.

## Longitudinal Design

Human interaction is sometimes fleeting, one word or two with a stranger without impact on the future, or so it seems. Other times, it spans over long time periods and even very long time periods. The history of events with people belonging to our social circles is fundamental to the definition of the self, and social bonds evolve continuously over time. A study by King-Casas et al. (2005) employed a paradigm of economic transaction across several rounds using fMRI. An investor had to decide how much money to give to the partner. The partner had to decide how to share the final benefit. The gain amounted to three times the initial investment. In the next round, the roles were reversed. Using a within- and cross-brain BOLD signal correlation measure, the authors found that when the relationship was made of trust, the magnitude of response in the caudate nucleus correlated with the intention of trusting in the following rounds. Additionally, while reputation was built between the participants over time, the trust signal occurred 14 s earlier in the next runs preceding the revelation of the investment. The temporal transfer of the trust signal shows that it shifted from reactive to anticipatory. Thus, the authors argued that the caudate nucleus activity reflects the development of a reputation for their partner through the construction of a model of the investor in the trustee’s brain. This study suggests that participants were able to form a mental representation of their partner and act accordingly in the next runs with concurrent correlates in brain structures. This is an adaptive behavior in reward prediction as seen in reinforcement learning models when interacting with a fellow human (Berridge, 2000). Structural brain changes go in parallel with behavioral modifications, as established for example in music training. In a study with 6 years-old children, significant structural brain changes have been ascertained after 15 months of weekly keyboard lessons, as well as improvement in musically relevant motor and auditory skills (Hyde et al., 2009). What is more, research has found that music practicing greatly improves the development of personal and social skills in children (i.e., higher self-esteem, increased motivation and self-efficacy), and thus have a positive effect over time (Hallam, 2010). Several researchers have theorized about the social skills and brain mechanisms developed in early life. Di Paolo and De Jaegher (2012) discussed about two components of their interaction brain hypothesis (IBH): contemporaneous (CIBH) and developmental (DIBH). The DIBH is described as following: “*The functions of individual brain mechanisms involved in social understanding have been shaped during development by skillful engagements in social interactions where interactive processes have been involved in social performance in a more than contextual way.*” This theoretical view implies that during social interactions brain mechanisms evolve and mature over time. Therefore, carrying out social interaction experiments with a young population could increase the chance to find neurophysiological changes; as a matter of fact, only longitudinal studies can uncover changes over time on the same participant. Besides, they present two advantages: they naturally favor ecological validity, since everyday social interaction often spans over long periods, and they enable the correlation of changes in behavioral performance and objective brain coupling phenomena over time.

## Music Performance

**FIGURE 4:**
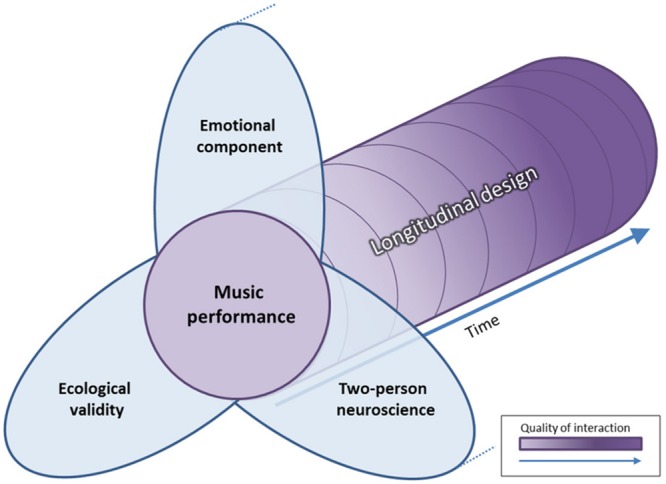
**Benefits of music performance over time as an experimental design for social interaction in neuroscience.** Three fundamental characteristics have been advised by authors in social neurosciences when it comes to studying social interactions. First, ecological validity assume that stimuli and experimental environment must mirrors as closely as possible real life. Second, the emotional component has been found to be a catalyst for social interaction and promotes brain coupling. Thirdly, the two-person neuroscience promotes an enactive view of human interaction rather than the observation paradigm, standard in classical neuroscience. At the crossroad of these three notions stands music performance. We argue that with the use of longitudinal design and the appropriate neuroimaging modality, it would be possible to describe the steps in building a musical relationship across several dimensions (e.g., behavioral, emotional, and neuronal). Hence, the quality of human interaction in these dimensions may improve (e.g., better performance, feeling of togetherness, and change in neural network) over time.

Designing an experiment with satisfying balance between ecological validity and experimental control, using a longitudinal design, in a continuous interaction and promoting emotional contagion can be challenging. Nevertheless, there is one particular human activity that could fulfill the criteria: *music performance* (**Figure 4**).

It has been argued that musicality is a fundamental part of being human, that this capacity is a very old evolutionary characteristic, and that a holistic protolanguage of musical emotive expression predates language and was an essential precursor to it (Mithen, 2005). Indeed, not only birds, but also whales, dolphins, old and new world monkeys, and apes sing to communicate with each other (Hauser and McDermott, 2003). Music has been considered important in social interaction for several reasons, also known as the 7 C-s. It is involved in social contact, enhances social cognition, permits co-pathy, communication, coordination of actions, cooperation and leads to group cohesion (Koelsch, 2014). Music is infused with the power to make us travel through the ages (Jäncke, 2008); it awakens our senses from an early age and marks our childhood memories. These social aspects of music combined with the potential of music to evoke emotions makes music a valuable tool for the investigation of social emotions and their neural correlates (Koelsch, 2014).

Music has been a prolific area of research in neuroscience in recent decades, investigating especially the related motor and auditory function and auditory perception (Zatorre et al., 2007), memory (Chan et al., 1998), language (Patel, 2003), and emotions (Blood and Zatorre, 2001). The neuroscience of music listening is a rich and growing field that relies on a large body of literature on neural mechanisms of music processing, ranging from cerebral injuries to studies with healthy participants using various modalities of investigations (Ayotte et al., 2000; Bhattacharya et al., 2001; Di Pietro et al., 2004; Meister et al., 2004). More importantly here, music performing possesses interesting properties for the investigation of social interaction. For instance, it involves three elementary motor control functions: timing, sequencing, and spatial organization of motor movements (Zatorre and Halpern, 2005). Timing and rhythm are crucial component of music performance as variation across these dimensions describes very different musical styles. Repetitions of rhythmical patterns, such as verses in a song structure, are typical in music productions. This provides an excellent frame for controlled experiment as repetition is a requirement for enhancing the signal-to-noise ratio of EEG data and/or power in inferential statistical analysis. Even in group improvisation, a condition where every musician has to cooperate to produce a collective, coherent and emergent musical construction without prior learning, there are some rhythmical rules to follow [chord progression, harmonic rhythm (Russo, 1997; Swain, 2002; Randel, 2003)]. Musical improvisation is an interesting paradigm when studying the neural basis of social interaction also because it requires constant rearrangement and cooperation between musicians to create something harmonious. Di Paolo and De Jaegher (2012) argue that a particularly interesting notion in coordination is *transitions*. Human interaction lies in coordination breakdowns and recoveries, the oscillation between these two states and the joint sense-making where the actions and intentions are co-constructed can be typically observed during musical performance, especially in improvisation. Musical harmony in fact depends on our auditory feedback. “Feedback” is when motor action precedes its auditory perception, and is especially relevant in tasks where musical production must be continuously controlled (e.g., improvisation or singing). If auditory feedback is blocked, musicians can still execute a previously memorized and well-rehearsed piece, but at the expense of emotional rendering (Repp, 1999). Auditory feedback is one of the two classes of auditory-motor interaction, the other one being “feedforward interaction.” The auditory system influences the motor output in a predictive way, for example when a listener taps his or her foot to the beat, anticipating the rhythmic accents in a musical production (Large and Palmer, 2002). This is another interesting property of music performance as the auditor has to *learn* the right pace.

Knowing how to play an instrument and how to play in a band is not innate. It must be learned by regularly practicing alone and with other musicians. Repeating the process in time enables musicians to refine their technique, driving changes in brain anatomy and physiology (Münte et al., 2002). Playing music does not involve only a passive audition; instead it is an engaging multimodal social task. Playing an instrument is accompanied by peculiar movements, often of the whole body, which naturally solicit movement and expression in other players, singers, and dancers. This positive urge to move along with music is referred to “being in groove.” The amount of experienced groove and social enjoyment is greater when sensorimotor coupling with music is better and more easily achieved with others (Janata et al., 2012; Fairhurst et al., 2013). The resulting social, physical, and emotional contagion enhance social experiences such as learning, imitation, shared understanding, laughter, feeling of togetherness, making of music performance a powerful catalyzer for social relationships. According to the Shared Affective Motion Experience (SAME model) the musical sound bears cognitive and emotional information for the action of generating music (Overy and Molnar-Szakacs, 2009). Emotions are tightly linked to music listening and production (Juslin and Sloboda, 2001). The ability of music to convey different emotional states is known to develop early in childhood, and improves significantly through development (Dowling, 1999). As music may genuinely evoke emotional responses without experimental deceit, we can consider it to be an excellent task to explore social interaction. In particular, the possibility to learn a music partition over long period of time may be a convenient way to design longitudinal experiments. Additionally, this activity is fully compatible with current imaging modalities such as EEG, fNIRS and fMRI (Blood and Zatorre, 2001; Müller et al., 2013).

## Discussion and Conclusion

The goal of social neuroscience is to understand the neural underpinning of social interaction. This requires the study of humans in free interaction (Hari and Kujala, 2009; Di Paolo and De Jaegher, 2012). In this paper, we have reviewed EEG hyperscanning studies exploring continuous interactions (**Table 1**). Two consistent observations can be drawn from these studies. First, it has been shown that there is an inter-subject asymmetry in EEG power, mostly in the alpha (8–12 Hz) band in frontal electrodes, when examining leader-follower interactions. The suppression of the leader’s alpha power might reflect self-allocation of resources, but may also be related to empathy, especially in the case of the follower (Babiloni et al., 2012; Konvalinka et al., 2014). Second, there seems to be two networks at play during social interaction: an intra-individual one oscillating at higher frequencies such as alpha (8–14 Hz) and beta (14–28 Hz), and an inter-individual one oscillating at lower frequencies such as delta (1–4 Hz) and theta (4–7 Hz). The latter may be more difficult to study as its emergence is likely more transient and fragile. To increase the occurrence of synchronized events and reinforce their stability, we propose to elicit emotions as they are a catalyst of social interaction and appear to promote brain coupling across participants (Nummenmaa et al., 2012; Kawasaki et al., 2013). We have also discussed the relevance of longitudinal designs to increase the chance to uncover inter-brain phase synchronization phenomena as they may appear on the basis of mutual knowledge over time. To our knowledge, no hyperscanning studies have employed a longitudinal design yet. Doing so may help understanding how brain networks mature as the relationship between people builds up. We have also stressed the importance of the ecological characteristic of the experiments as we think that this is a key feature when investigating human interactions. Based on these premises, we have concluded that music performance is a suitable experimental paradigm for hyperscanning studies. Indeed, a music performance requires fine cognitive abilities, is possible only thanks to a continuous feedback received from the partners and is intimately related to emotions. Our arguments flourish in the context of several recent theoretical and experimental studies that have been employed successfully music performance (Lindenberger et al., 2009; Overy and Molnar-Szakacs, 2009; Babiloni et al., 2012; Müller et al., 2013; D’Ausilio et al., 2015; Hunt, 2015). One may argue that a musical performance requires highly specialized skills disabling the generalization to the general population. Indeed, research has shown that some brain structures of musicians significantly differ from those of non-musicians, especially in motor, auditory and visuo-spatial regions due at least in part to structural adaptations in response to long-term skill acquisition and their training (Gaser and Schlaug, 2003). However, most human beings share an implicit musical ability and therefore possess basic skills that are comparable to the musicians when it comes to listening to musical performances (Koelsch et al., 2000). Thus future findings on social interaction between musicians may somehow extend to the rest of the population and may not be specific to musicians. A recent opinion by Dumas et al. (2014) presented music as well as dance performance as activity providing simultaneously ecological situations linking the first (subjective: feelings, emotions) and third (objective: physiological measures) person perspective. This view supports our claim on music performance stressing the notion that researchers should consider the subjective experience of the relationship (Dumas et al., 2014). Regarding other ecological activities, dance and singing share the same emotional engagement as music performance, which make them interesting to study. For example, Müller and Lindenberger (2011) have observed an oscillary coupling of respiratory and cardiac activity between conductor and singers in a choir. The experiment carried out by Bachrach et al. (2015) studied audience-dancers coordination on physiological (respiration), cognitive (time perception) and subjective engagement levels during a contemporary dance performance. The authors found that the degree of the synchronization between observer and dancer was associated with the attention of the spectator to breathing. This pilot study shows the feasibility to record the physiological responses of dancers while they are performing (Bachrach et al., 2015). However, dancing, singing and doing sports (i.e., synchronized rowing) is expected to engender more movement and muscular artifacts in the EEG recordings. As suggested by Dumas et al. (2014), future neuroimaging modalities and experimental protocols could adress some of these concerns. EEG excels at recording very quick changes in brain rhythms, however, it falls short to decypher slow dynamics found in turn-based social interactions. Moreover, EEG lacks a good spatial resolution, which is of paramount importance to pinpoint the brain structures involved in the activity under study. As Koike et al. (2015) argued, a multi-modal investigation may help improving the spatial and temporal resolution. They promoted the use of EEG-fMRI hyperscanning. However, as we pointed out earlier, ecological situations are crucial when conducting experiments on social interaction and fMRI does not allow them. A suitable option appears a joint EEG and fNIRS (functional near-infrared spectroscopy) modality; the spatial and temporal characteristics of fNIRS are intermediate between those of fMRI and EEG, and fNIRS allows the same ecological validity as EEG (Cui et al., 2011; Koike et al., 2015). Furthermore, fNIRS may be more suitable than EEG for grasping the slow dynamics that are at play in turn-based interactions.

In conclusion, the last decade has witnessed a bloom in social neurosciences. This new field of research calls for a paradigm shift. Based on recents findings, we argue that music performance is a suitable experimental paradigm to study human interaction and co-operation.

## Author Contributions

All authors listed, have made substantial, direct and intellectual contribution to the work, and approved it for publication.

## Conflict of Interest Statement

The authors declare that the research was conducted in the absence of any commercial or financial relationships that could be construed as a potential conflict of interest.
